# Different Expressions of Specific Transcription Factors of Th1 (*T-bet*) and Th2 cells (*GATA-3*) by Peripheral Blood Mononuclear Cells From Patients With Multiple Sclerosis

**DOI:** 10.32598/bcn.9.6.458

**Published:** 2018-11-01

**Authors:** Zahra Etesam, Maryam Nemati, Mohammad-Amin Ebrahimizadeh, Hossain-Ali Ebrahimi, Hossain Hajghani, Tahereh Khalili, Abdollah Jafarzadeh

**Affiliations:** 1. Neurology Research Center, Kerman University of Medical Sciences, Kerman, Iran.; 2. Department of Immunology, Faculty of Medicine, Kerman University of Medical Sciences, Kerman, Iran.; 3. Department of Hematology and Laboratory Sciences, School of Para-Medical, Kerman University of Medical Sciences, Kerman, Iran.; 4. Department of Biochemistry, Faculty of Medicine, Kerman University of Medical Sciences, Kerman, Iran.; 5. Molecular Medicine Research Center, Rafsanjan University of Medical Sciences, Rafsanjan, Iran.; 6. Department of Immunology, School of Medical, Rafsanjan University of Medical Sciences, Rafsanjan, Iran.

**Keywords:** Multiple Sclerosis, Th1, Th2, *T-bet*, *GATA-3*

## Abstract

**Introduction:**

Multiple Sclerosis (MS) is an inflammatory disorder caused by self-reactive Th1 lymphocytes, while Th2 cells may confer protection. The Th1 and Th2 cell differentiation are regulated by specific transcription factors, especially *T-bet* and *GATA-3*, respectively. This investigation aimed to measure the *T-bet* and *GATA-3* expression by Peripheral Blood Mononuclear Cells (PBMCs) obtained from MS patients after specific and non-specific in vitro stimulation.

**Methods:**

The PBMCs were separated from 22 patients with MS and 20 healthy individuals. They were cultured at 37°C for 24 h in the absence of a stimulator or in the presence of Myelin oligodendrocyte Glycoprotein (MOG) or Phytohemagglutinin (PHA) at a concentration of 10 μg/mL. Then the *T-bet* and *GATA-3* expression was measured by real time-PCR.

**Results:**

The *T-bet* expression was enhanced, while the *GATA-3* expression diminished. Therefore the expression of *T-bet*/*GATA-3* ratio diminished in not-stimulated, MOG-stimulated and PHA-stimulated PBMCs from MS patients compared with equal cultures from the healthy individuals (P<0.01, P<0.01 and P<0.01, for *T-bet*; P<0.03, P<0.01 and P<0.02, for *GATA-3*; P<0.01, P<0.001 and P<0.01 for *T-bet*/*GATA-3* ratio, respectively). The not-stimulated, MOG-stimulated, and PHA-stimulated PBMCs from men with MS expressed higher amounts of *GATA-3* than equal cells from MS women (P<0.05, P<0.05 and P<0.01, respectively).

**Conclusion:**

These results probably indicate an imbalance in Th1/Th2 cells in the level of transcription factors with a tendency toward Th1 cells in MS. The clinical utilization of the transcription factors as novel biomarkers of MS should be evaluated in further studies.

## Highlights

1. Multiple Sclerosis (MS) is an inflammatory disorder caused by self-reactive Th1 lymphocytes, while Th2 cells may confer protection.
2. The Th1 and Th2 cell differentiation are regulated by specific transcription factors, especially *T-bet* and *GATA-3*, respectively.
3. We measured the *T-bet* and *GATA-3* expression by Peripheral Blood Mononuclear Cells (PBMCs) obtained from MS patients.
4. The *T-bet* expression was enhanced, while the *GATA-3* expression weakened.
5. The expression of *T-bet/GATA-3* ratio was lower in not-stimulated, MOG-stimulated and PHA-stimulated PBMCs from MS patients compared with equal cultures from the healthy individuals.
6. The not-stimulated, MOG-stimulated, and PHA-stimulated PBMCs from men with MS expressed higher amounts of *GATA-3* than the same cells from MS women.
7. These results probably indicate an imbalance in Th1/Th2 cells in the level of transcription factors with a tendency toward Th1 cells in MS.

## Plain Language Summary

Multiple Sclerosis (MS) is an inflammatory disorder caused by self-reactive Th1 lymphocytes, while Th2 cells may confer protection. This research aimed to measure the *T-bet* and *GATA-3* expression by Peripheral Blood Mononuclear Cells (PBMCs) obtained from MS patients. The PBMCs were separated from 22 patients with MS and 20 healthy individuals. The *T-bet* expression was enhanced, while the *GATA-3* expression weakened. Therefore the expression of *T-bet/GATA-3* ratio diminished in not-stimulated, MOG-stimulated and PHA-stimulated PBMCs from MS patients compared with equal cultures from the healthy individuals. The not-stimulated, MOG-stimulated, and PHA-stimulated PBMCs from men with MS expressed higher amounts of *GATA-3* than equal cells from MS women. These results probably indicate an imbalance in Th1/Th2 cells in the level of transcription factors with a tendency toward Th1 cells in MS. The clinical implication of the transcription factors as novel biomarkers of MS should be evaluated in further studies.

## 1. Introduction

Multiple Sclerosis (MS) results in the demyelination of neurons within the brain and spinal cord due to inflammatory and autoimmune reactions (Mahad Trapp & Lassmann, 2015). Its prevalence is reported as 57.5 per 100000 people in Kerman City, Iran (Ebrahimi & Sedighi, 2013). The MS-related pathological events are mainly attributed to the activation of autoreactive CD4+ T helper (Th) cells (Kostic, Stojanovic, Marjanovic, Zivkovic, & Cvetanovic, 2015). Functionally, the effector CD4+ T-cells are classified into several subgroups such as Th1, Th2, Th17, or regulatory T (Treg) cells differentiated from naive T-cells after antigenic recognition in the presence of particular cytokines (Raphael, Nalawade, Eagar, & Forsthuber, 2015).

The Interferon (IFN)-γ-secreting Th1 cells and Inter-leukin (IL)-17-producing Th17 cells are involved in the MS- and experimental autoimmune encephalomyelitis (EAE)-related pathological events (Etesam et al., 2016; Jafarzadeh et al., 2015a; Raphael et al., 2015). Treg cell-related cytokines (Transforming growth factor β, IL-10, and IL-35) are associated with a reduction of Central Nervous System (CNS) inflammation and improvement of MS and EAE symptoms (Jafarzadeh et al., 2017; Jafarzadeh et al., 2015b; Raphael et al., 2015). The contradictory roles were attributed to Th2 cells, which secrete high amounts of IL-4, IL-5, and IL-13 (Raphael et al., 2015).

Elevated amounts of CCL20 (a Th17-linked chemokine) and diminished amounts of CCL22 (a Th2/Treg-linked chemokine) were observed in patients with MS (Jafarzadeh et al., 2014a; Jafarzadeh et al., 2014b). Elevated expression of IL-33 (a Th2-linked cytokine) was also observed in MS and EAE diseases (Jafarzadeh et al., 2016; Jafarzadeh et al., 2014c).

The specific cellular transcription factors of *T-bet* and *GATA-3* control the effector Th1 and Th2 cell differentiation from naïve T-lymphocytes, respectively (Zhang et al., 2014b). IFN-γ and IL-12 cause Th1 cell differentiation via activating STAT-1 (signal transducer and activator of transcription-1) and STAT-4 (Zhang et al., 2014b). Both STAT-1 and STAT-4 cause *T-bet* (T box expressed in T-cells) expression, which operate as a Th1 cell-related major transcription factor. *T-bet* induces IFN-γ production, which also reinforces the Th1 cell polarization (Zhang et al., 2014b). IFN-γ-producing Th1 cells mediate the cellular immunity that performs a major role in defense against intracellular pathogens and is also involved in the pathological process of some autoimmune disorders (Zhang et al., 2014a).

IL-4 (initially synthesized by mast cells and basophils) induces Th2 cell differentiation via activating STAT-6 (Na et al., 2016). STAT-6 causes the *GATA-3* (GATA binding protein 3) expression, which operates as a Th2 cell-related major transcription factor (Na et al., 2016). *GATA-3* induces the IL-4, IL-5, and IL-13 production providing a self-reinforcing feedback loop (Zhang et al., 2014b). Th2 cells mediate the humoral immune responses, which play a crucial role in defending extracellular infectious agents and are also involved in the pathogenesis of a number of autoimmune diseases (Na, Cho, & Chung, 2016; Zhang et al., 2014a).

It is noteworthy that the Th1/Th2 cells functionally antagonize each other. For instance, the *T-bet* production is suppressed by STAT-6, whereas *GATA-3* synthesis is directly inhibited by *T-bet* (Evans & Jenner, 2013). The development of a number of immunopathological responses are attributed to the imbalance between Th1 and Th2 cell activation (Zhang et al., 2014a). The determination of the *T-bet*/*GATA-3* ratio may be more reliable than the assessment of a single Th1 or Th2 cell-linked parameter regarding the determination of Th1/Th2 cell balance (Lin et al., 2015). Thus, studying the Th1/Th2 cell balance at the level of their transcription factors reveals more important information.

There are a number of reports on the patients with MS or on EAE models concerning the determination of some transcription factors (Edstrom et al., 2011; Martinez et al., 2014); however, there are no data about the assessment of these elements in PBMCs from patients with MS following specific and non-specific stimulations. Thus, the current study aimed at determining the gene expression of *T-bet* and *GATA-3* and their ratio by PBMCs obtained from newly-diagnosed patients with MS following the in vitro stimulation with Myelin Oligodendrocyte Glycoprotein (MOG), Phytohemagglutinin (PHA), or without stimulation to identify any associations.

## 2. Methods

### 2.1. Subjects

The participants were 22 newly-diagnosed patients with MS (8 men and 14 women) and 20 healthy individuals (8 men and 12 women). The patients were admitted to the MS Center of Shephah Hospital affiliated to Kerman University of Medical Sciences (Kerman, Iran) and expressed RRMS (Relapsing-remitting MS) pattern of the disease. The McDonald’s criteria (McDonald et al., 2001) using clinical and paraclinical findings (MRI observations, oligoclonal bands in Cerebral Spinal Fluid (CSF) and evoked potentials) were employed to diagnose MS.

The healthy individuals were recruited among the blood donors of the local Kerman Blood Transfusion Center, and matched to the patients regarding gender and age. The healthy individuals were in good health conditions, without medical history of CNS related diseases, recurrent infections, immunological disorders, malignancy, asthma, allergy and or atopic diseases. History of using medication, smoking, operation, and severe trauma within six months prior to blood collection were among other exclusion criteria. A sample of peripheral blood (5 mL) was collected from each participant and their PMBCs were isolated for more analysis.

### 2.2. In vitro stimulation of PBMCs

A gradient centrifugation method over the LymphoSep (Biosera, UK) was employed to isolate PBMCs from the heparinized peripheral blood. The PBMCs layer was carefully harvested and washed three times with Roswell Park Memorial Institute (RPMI)-1640 medium. Then, the PBMCs were again suspended in the supplemented-RPMI-1640 medium (a medium contained 10% heat inactivated fetal bovine serum [Gibco Life Technologies Ltd, Paisley, UK], 100 U/mL of penicillin, and 100 μg/mL of streptomycin).

The PBMCs were then dispensed in the 24-well sterile flat-bottomed microtiter plates (1×10^6^ cell/well) and cultured (at 37°C in a 5% CO2 incubator for 24 hours) in the absence of a stimulator, in the presence of MOG (35–55) human (Anaspec, USA) or PHA (Gibco Life Technologies Ltd, Paisley, UK) at a concentration of 10 μg/mL. After this time, the total RNA was extracted from the PBMCs for more analyses.

### 2.3. RNA extraction, reverse transcription, and quantitative real-time PCR

The Trizol reagent (Bionner, Korea) was used to extract the total RNA from cultured PBMCs. Then, the extracted RNA was treated with DNase I (Thermo Scientific, EU) to eliminate the possible contamination with genomic DNA. The purity of the extracted RNA was assessed by electrophoresis on the agarose gel (pretreated with ethidium bromide) along with the calculation of 260/280 absorption ratio by a spectrophotometer system.

The conversion of the extracted RNA into complementary DNA (cDNA) was performed using a cDNA synthesis kit (Bionner, Korea), which contained both oligo (dT) and random hexamer primers. The *T-bet* and *GATA-3* expression was estimated using a real-time Polymerase Chain Reaction (PCR) technique. The *β-actin* gene was also employed as an internal control. The employed primers were purchased from Bionner Company (Korea).

Real-time PCR procedure was performed using a real-time PCR system (Applied Biosystems, USA) in a triplicate manner using a SYBR green master mix (Bionner, Korea) combined with 2 μL of appropriate primers (Table 1) and 200 ng of template cDNA. The protocol of reverse transcription was an initial heating at 95°C for 15 minutes (in the absence of reverse transcriptase enzyme), −20°C for 60 seconds (cooling phase), adding reverse transcriptase, 40 consecutive cycles of 95°C for 30 seconds and 60°C for 30 seconds, and a final 72°C for 30 seconds.

**Table 1:** The sequence of primers used to assess *T-bet* and *GATA-3* expression by PBMCs in the MS and control groups

**Gene**	**Primer**
*T-bet*	F: 5-GGGAAACGGATGAAGGACTGAGA-3 R: 5-TTAGGGCAGAGGATGGGGCAA-3
*GATA-3*	F: 5-TCATTAAGCCCAAGCGAAGG-3 R: 5-GTCCCCATTGGCATTCCT-3
*β-Actin*	F: 5-GCATGGGTCAGAAGGATTC-3 R: 5-GTCCCAGTTGGTGACGAT-3

The *β-actin* gene, as a housekeeping gene, was employed to normalize the amplified *T-bet* and *GATA-3* genes. The amounts of the *T-bet* and *GATA-3* expression in the PBMCs were calculated by the 2^−ΔΔCt^ formula. The Applied Biosystems software version 1.1.308.111 (USA) was also employed to analyze the melting curves and the quantitative assessment of the data. The PCR products were also visualized on a 1% agarose gel (containing 0.5 mg/mL ethidium bromide) following electrophoresis.

### 2.4. Statistical analysis

The results were expressed as Mean±SEM. The comparison of the variables was performed using appropriate statistical tests including ANOVA, t test, the Kruskal-Wallis, and the Mann-Whitney U test. P values less than 0.05 were also considered significant.

## 3. Results

The demographic and baseline characteristics of participants are presented in Table 2. The differences of the age and gender ratio between the MS and healthy groups were not significant (P=0.79 and P=0.80, respectively).

**Table 2:** Demographic and baseline characteristics of the study participants

**Characteristics**		**Group**	

		**MS (n=22)**	**Control (n=20)**
Age (Mean±SD, y)		35.56±11.16	36.45±11.73
Gender	Male	8(36.37%)	8(40.00%)
	Female	14(63.63%)	12(60.00%)
Medications		0	0
Current smoking		0	0
Disabling symptoms	Muscle weakness	14(63.63%)	0
	Muscle spasm	5(22.72%)	0
	Ocular problems	6(27.27%)	0
	Urinary incontinence	6(27.27%)	0
	Fatigue	5(22.72%)	0
	Lack of balance	6(27.27%)	0
	Dysarthria	3(13.63%)	0
	Headache	4(18.18%)	0

### 3.1. The *T-bet* and *GATA-3* expression in healthy individuals and patients with MS

The PHA-stimulated PBMCs from both healthy control and MS groups expressed higher amounts of *T-bet*, *GATA-3*, and *T-bet*/*GATA-3* ratio when compared with those of non-stimulated cultures (P<0.01, P<0.05, and P<0.05, respectively) (Table 3). The MOG-stimulated PBMCs of patients with MS expressed higher levels of *T-bet* as compared with those of non-stimulated cultures (P<0.05) (Table 3). In healthy individuals, the *T-bet* expression in MOG-stimulated PBMCs was also higher than that of non-stimulated cultures, but the difference was not significant.

**Table 3:** The *T-bet*, *GATA-3*, and *T-bet*/*GATA-3* expression ratio by PBMC in the MS and control groups

**Group**	**Stimulated PBMCs**	**Gender**	***T-bet* Expression**	***GATA-3* Expression**	***T-bet/GATA-3* mRNA Ratio**
Control	Without stimulation	Male	0.65±0.29	1.30±0.61	4.50±3.51
		Female	1.22±0.66	0.81±0.33	3.66±1.18
		Total	0.43	1.00±0.31	3.97±1.43
	MOG	Male	0.90±0.62	5.88±2.70	17.41±15.92
		Female	4.24±2.21	5.12±2.14	13.64±11.78
		Total	2.94±1.40	5.42±1.63	15.10±9.21
	PHA	Male	15.15±8.48	18.78±11.95	24.49±15.26
		Female	10.95±6.70	6.16±3.55	48.65±42.63
		Total	12.72±4.65	11.21±5.24	39.26±26.33
MS	Without stimulation	Male	4.78±3.43	0.52±0.46	40.21±37.50
		Female	8.30±4.79	0.05±0.008	88.17±54.59
		Total	6.89±3.09	0.24±0.18	70.18±35.94
	MOG	Male	22.62±14.07	1.29±0.83	33.83±24.01
		Female	21.23±7.70	0.38±0.20	188.93±71.26
		Total	21.72±6.80	0.67±0.30	130.77±48.49
	PHA	Male	25.81±13.68	6.69±3.55	100.01±55.99
		Female	32.74±11.49	1.05±0.59	718.09±371.90
		Total	30.32±8.69	2.75±1.23	453.20±225.77

In healthy individuals, the MOG-stimulated *GATA-3* expression in PBMCs was significantly enhanced in comparison with that of non-stimulated cultures (P<0.01). However, the *GATA-3* expression did not show significant difference between MOG-stimulated and non-stimulated PBMCs of patients with MS (Table 3). In the healthy control group and patients with MS, the differences in the expression of *T-bet*/*GATA-3* ratio between MOG-stimulated and non-stimulated PBMC were not statistically significant, although, this ratio was higher in MOG-stimulated PBMCs (Table 3).

**Figure 1:**
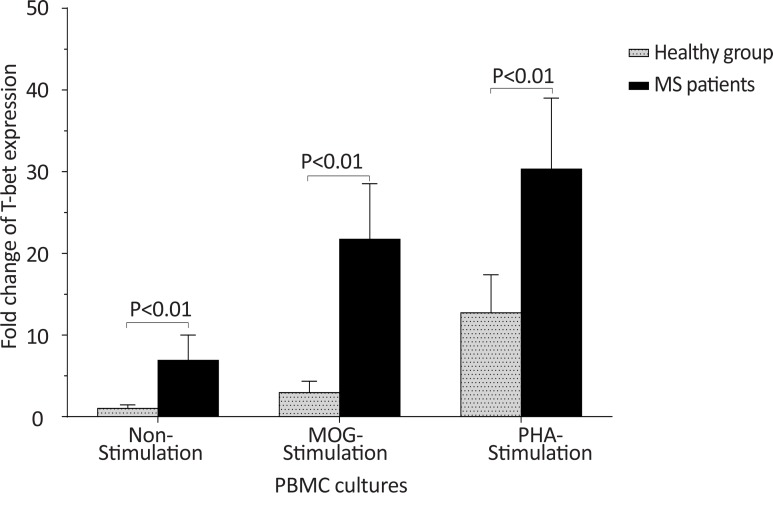
The comparison of *T-bet* expression by PBMC between the MS and control groups The non-stimulated, MOG-stimulated, and PHA-stimulated PBMCs from MS patients expressed higher amounts of *T-bet* in comparison with the same cell cultures from healthy individuals.

**Figure 2:**
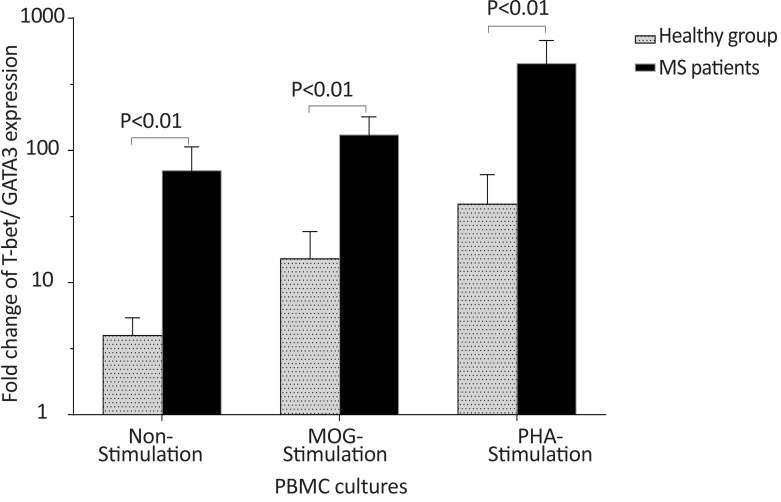
The comparison of *T-bet*/*GATA-3* expression ratio by PBMC between the MS and control groups The non-stimulated, MOG-stimulated, and PHA-stimulated PBMCs from MS patients expressed higher amounts of *T-bet*/*GATA-3* mRNA ratio in comparison with same cell cultures from healthy individuals.

**Figure 3:**
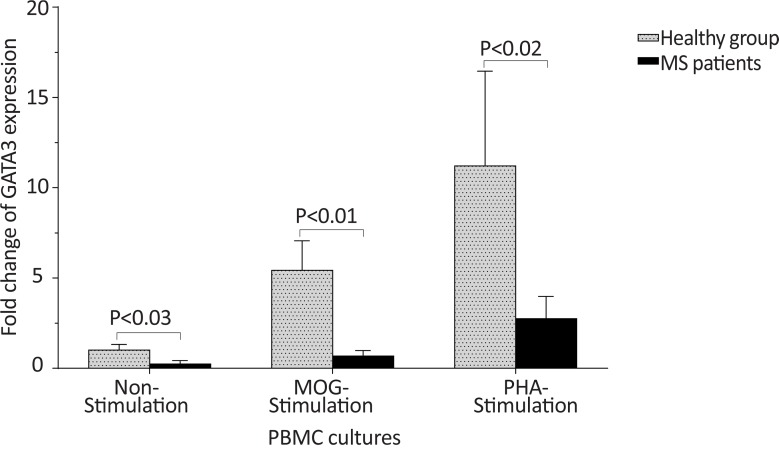
The comparison of *GATA-3* expression by PBMC between the MS and control groups The non-stimulated, MOG-simulated, and PHA-stimulated PBMCs from MS patients expressed lower amounts of *GATA-3* in comparison with same cell cultures from healthy individuals.

The *T-bet* expression and the *T-bet*/*GATA-3* ratio in PBMCs of patients with MS were higher than those of the counterpart cell cultures of the healthy individuals after stimulation with MOG, after stimulation with PHA, or in the absence of a stimulator (P<0.01, P<0.01, and P<0.01 for *T-bet*; P<0.01, P<0.001, and P<0.01 for *T-bet*/*GATA-3* ratio, respectively) (Figures 1 and 2). However, the PBMCs of patients with MS expressed lower levels of GATA3 after stimulation with MOG, after stimulation with PHA, or in the absence of a stimulator, than those of the equivalent cell cultures of the healthy individuals (P<0.03, P<0.01, and P<0.02, respectively) (Figure 3).

### 3.2. The *T-bet* and *GATA-3* expression according to the gender of participants

**Figure 4:**
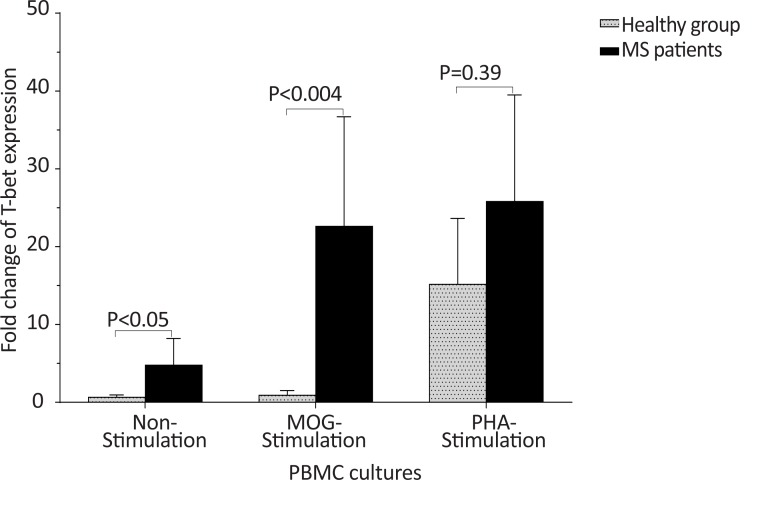
The comparison of *T-bet* expression by PBMC between healthy males and male patients with MS The expression of *T-bet* in non-stimulated and MOG-stimulated PBMCs from MS male patients were significantly higher than that of the same cultures from the healthy males (P<0.05 and P<0.004, respectively).

**Figure 5:**
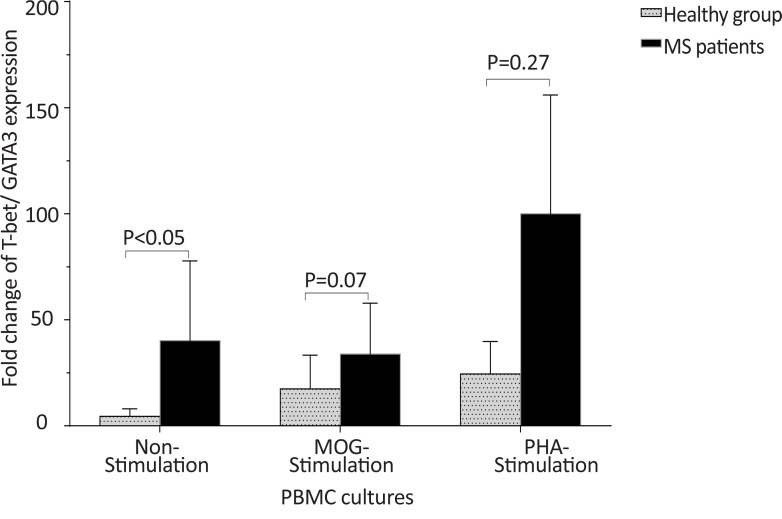
The comparison of *T-bet*/*GATA-3* mRNA ratio by PBMC between healthy men and male patients with MS The *T-bet*/*GATA-3* expression ratio in non-stimulated PBMCs from male patients with MS was significantly higher than that of the same culture from the healthy males. The *T-bet*/*GATA-3* expression ratio in MOG-stimulated PBMCs from male patients with MS was higher than that of the same culture from healthy males (P=0.07).

**Figure 6:**
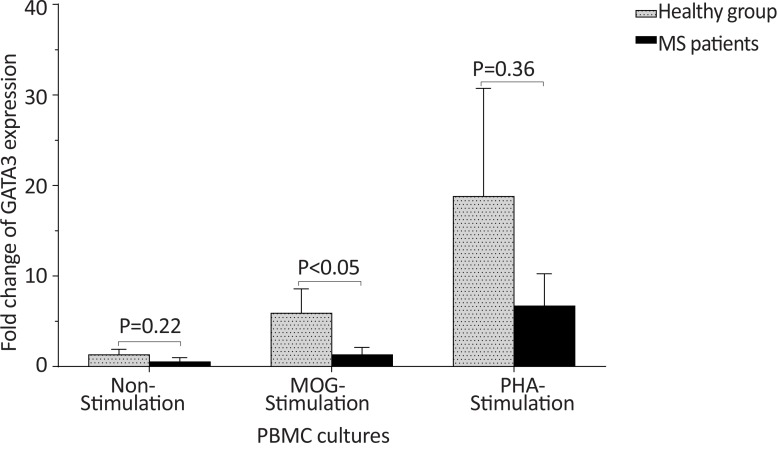
The comparison of *GATA-3* expression by PBMCs between healthy males and male patients with MS The *GATA-3* expression in MOG-stimulated PBMCs from males with MS was significantly lower than that of the same culture from healthy males. The *GATA-3* expression in non-stimulated and PHA-stimulated PBMCs from male patients with MS was lower than that of the same cultures from the healthy males, but the differences were insignificant.

The *T-bet* and *GATA-3* expression and the *T-bet*/*GATA-3* expression ratio in the control and MS groups, according to gender are summarized in Table 3. The males with MS exhibited higher *T-bet* expression in non-stimulated and MOG-stimulated PBMCs (P<0.05 and P<0.004, respectively), higher *T-bet*/*GATA-3* expression ratio in non-stimulated PBMCs (P<0.05), lower *GATA-3* expression in MOG-stimulated PBMCs (P<0.05) in comparison with counterpart cultures from healthy subjects with the same gender (Figures 4, 5, and 6).

**Figure 7:**
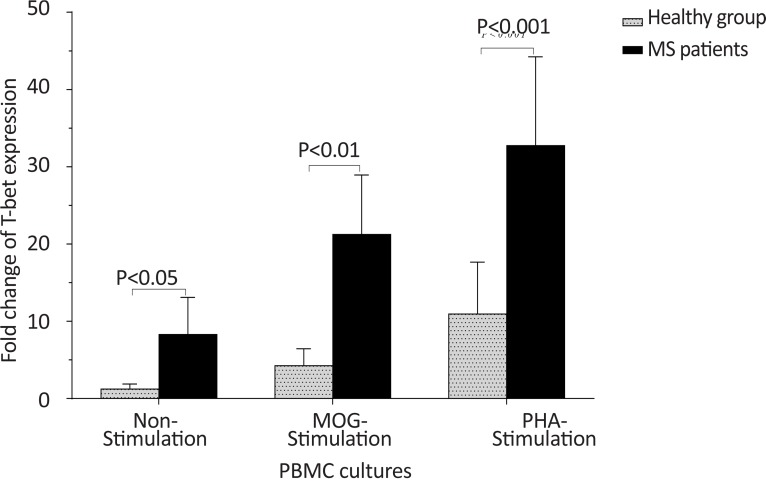
The comparison of *T-bet* expression by PBMCs between healthy females and female patients with MS The expression of *T-bet* in non-stimulated, MOG-stimulated, and PHA-stimulated PBMCs from female patients with MS was significantly higher than that of the same cell cultures from the healthy females.

**Figure 8:**
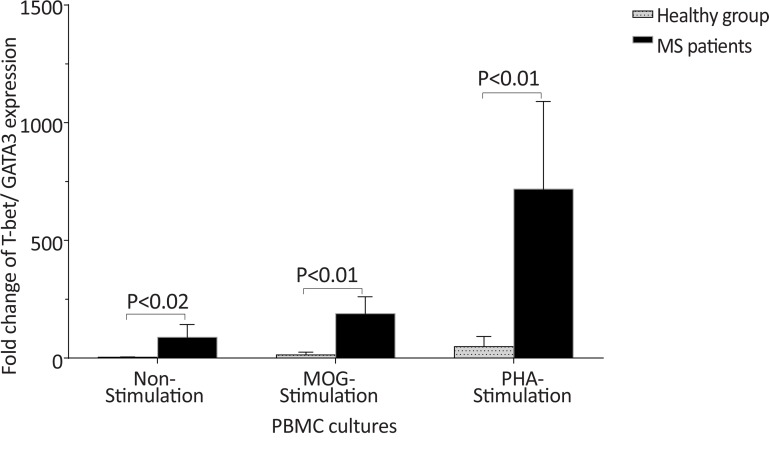
The comparison of *T-bet*/*GATA-3* mRNA Ratio by PBMCs between healthy females and female patients with MS The *T-bet*/*GATA-3* expression ratio in non-stimulated, MOG-stimulated, and PHA-stimulated PBMCs from female patients with MS was significantly higher than that of the same cell cultures from the healthy females.

**Figure 9:**
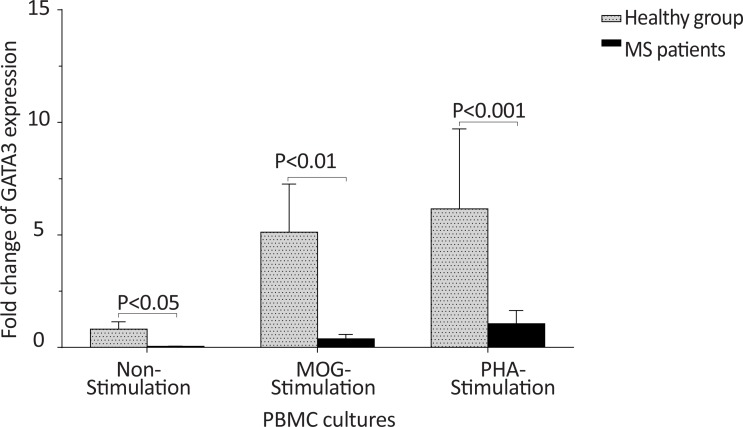
The comparison of *GATA-3* expression by PBMCs between healthy females and female patients with MS The *GATA-3* expression in non-stimulated, MOG-stimulated and PHA-stimulated PBMCs from MS female patients was significantly lower than that of the same cell cultures from the healthy females.

The females with MS also exhibited higher *T-bet* expression, higher *T-bet*/*GATA-3* expression ratio, and lower *GATA-3* expression in non-stimulated, MOG-stimulated, and PHA-stimulated PBMCs in comparison with equal cell cultures from healthy individuals with the same gender (P<0.05, P<0.01, and P<0.001 for *T-bet*; P<0.02, P<0.01, and P<0.01 for *T-bet*/*GATA-3* ratio; P<0.05, P<0.01, and P<0.001 for *GATA-3*, respectively) (Figures 7, 8, and 9).

**Figure 10:**
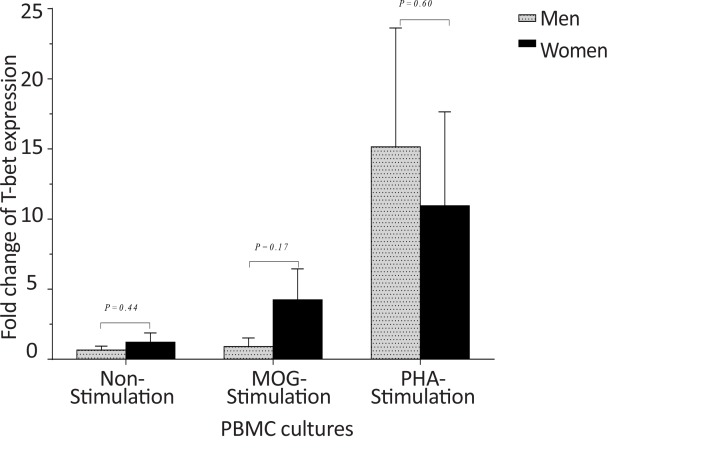
The Comparison of *T-bet* expression by PBMCs in healthy individuals, based on gender In non-stimulated, MOG-stimulated, and PHA-stimulated PBMCs, no significant differences were observed between healthy males and females in terms of *T-bet* expression.

**Figure 11:**
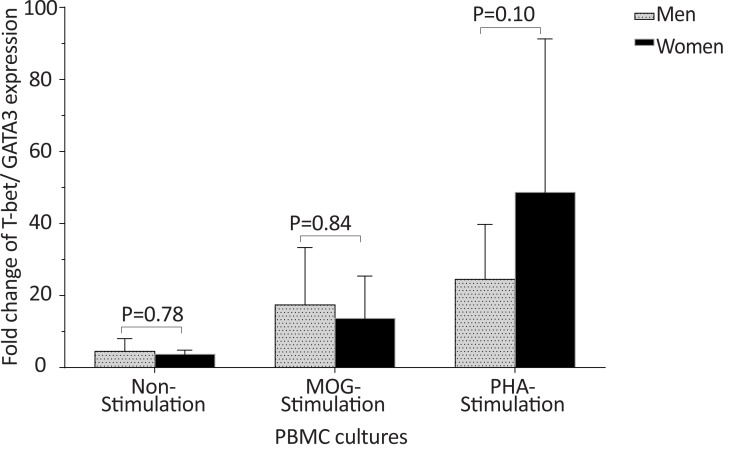
The comparison of *T-bet*/*GATA-3* mRNA ratio by PBMCs in healthy subjects, based on gender In non-stimulated, MOG-stimulated, and PHA-stimulated PBMCs from healthy controls, no significant differences were observed between males and females in terms of *T-bet*/*GATA-3* expression ratio.

**Figure 12:**
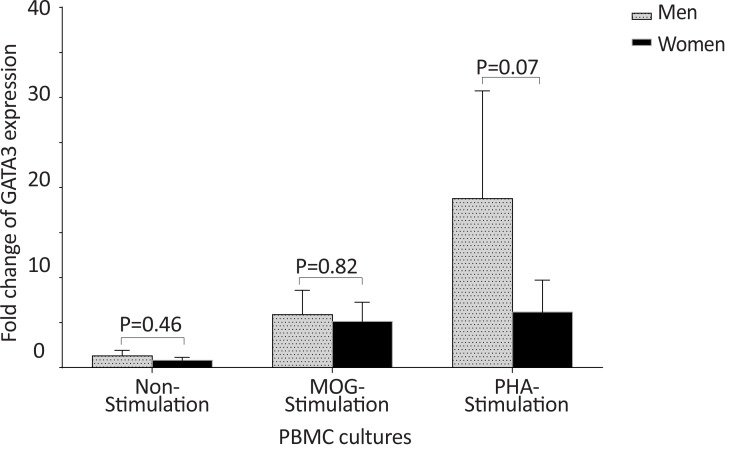
The comparison of *GATA-3* expression by PBMCs in healthy individuals, based on gender In non-stimulated, MOG-stimulated, and PHA-stimulated PBMCs from healthy controls, no significant differences were observed between males and females in terms of *GATA-3* expression.

**Figure 13:**
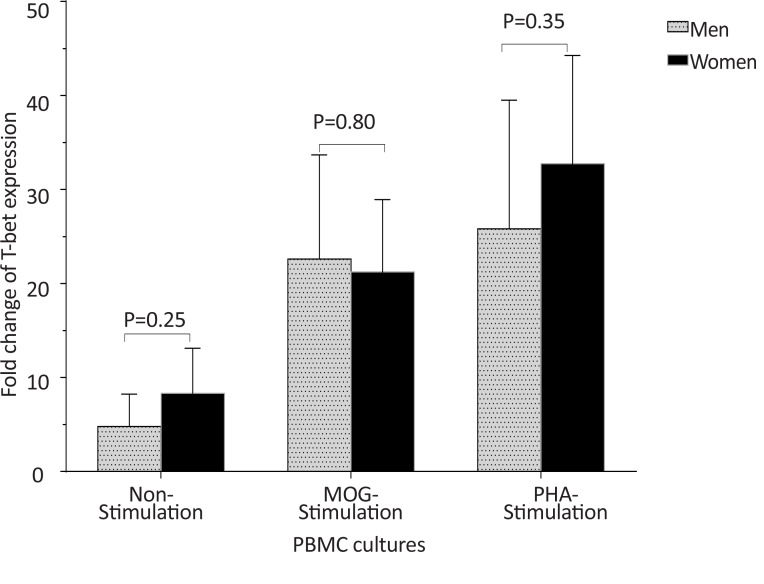
The comparison of *T-bet* expression by PBMCs in patients with MS, based on gender In non-stimulated, MOG-stimulated, and PHA-stimulated PBMCs from patients with MS, no differences were observed between males and females in terms of *T-bet* expression.

**Figure 14:**
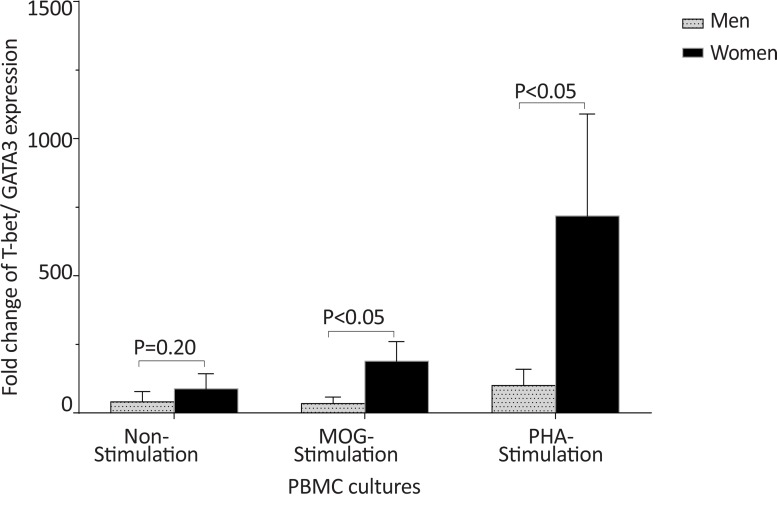
The comparison of *T-bet*/*GATA-3* mRNA ratio by PBMCs in patients with MS, based on gender The of *T-bet*/*GATA-3* expression ratio in MOG-stimulated and PHA-stimulated PBMCs from females with MS were significantly higher than that of the same cultures from male patients with MS.

In healthy individuals, the *T-bet* and *GATA-3* expression, and the bet/*GATA-3* expression ratio did not significantly differ between males and females in the three cultures, including non-stimulated, MOG-stimulated, and PHA-stimulated PBMCs (Figures 10, 11, and 12). No significant differences were observed between male and female patients with MS concerning the *T-bet* expression by non-stimulated, MOG-stimulated, and PHA-stimulated PBMCs, although this parameter was higher in females than males (Figure 13). However, the *T-bet*/*GATA-3* expression ratio in MOG-stimulated and PHA-stimulated PBMCs of females with MS were significantly higher than those of the equivalent cultures from males with MS (P<0.05 and P<0.05, respectively) (Figure 14).

**Figure 15:**
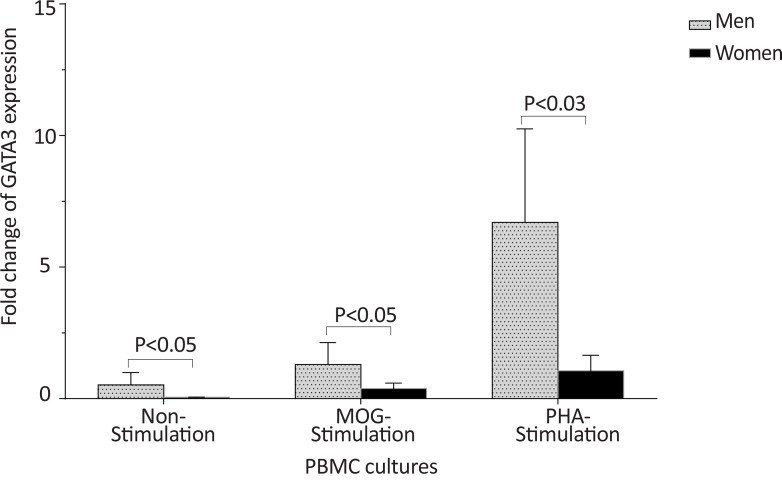
The comparison of *GATA-3* expression by PBMC in patients with MS, based on gender. The of *GATA-3* expression in non-stimulated, MOG-stimulated, and PHA-stimulated PBMCs from male patients with MS were significantly higher than that of the same cell cultures from female patients with MS.

The *GATA-3* expression in non-stimulated, MOG-stimulated, and PHA-stimulated PBMCs of males with MS were also significantly higher than those of equal cell cultures in females with MS (P<0.05, P<0.05, and P<0.01, respectively) (Figure 15).

## 4. Discussion

The current study results showed that the PHA-stimulated PBMCs of the healthy individuals and patients with MS expressed higher amounts of *T-bet* and *GATA-3* compared with those of their non-stimulated cultures. Different effector T-cells such as Th1 and Th2 cells are differentiated from naïve T-cells after exposure to PHA that operates as a powerful polyclonal T-cell inducer.

In healthy individuals, no significant difference was observed between non-stimulated and MOG-stimulated PBMCs, with respect to the *T-bet* expression while MOG-stimulated PBMCs of the patients with MS expressed higher amounts of this element compared with those of non-stimulated cultures. In patients with MS, the previous in vivo sensitivity of T-lymphocytes against myelin-related antigens (such as MOG) may be accountable for this difference.

The non-stimulated, MOG-stimulated, and PHA-stimulated PBMCs of patients with MS expressed higher levels of *T-bet* in comparison with those of equal cell cultures from the healthy individuals. The *T-bet* expression is rapidly induced in the early development of Th1 cells (Zhang et al., 2014b). These results reveal that the number of MOG-specific T-cells may be higher in patients with MS than the controls. Moreover, the higher *T-bet* expression by PHA-stimulated PBMCs of patients with MS represents more potential to generate Th1 cell-related immune responses in such subjects.

In accordance with the current study findings, it was postulated that Th1 cells reinforce the immunopathology in MS and EAE diseases, by releasing IFN-γ that has a crucial role in supporting autoimmunity (Hirahara & Nakayama, 2016). Importantly, *T-bet*-deficient mice were protected against EAE induction (Hirahara & Nakayama, 2016). Increased IFN-γ and TNF-α levels in the patients with MS further support that Th1 cells were pathogenic (Kallaur et al., 2013; Uysal, Meric Yilmaz, Bogdaycioglu, & Mungan Ozturk, 2014). It was also found that the Myelin Basic Protein (MPB)-specific T-lymphocytes separated from the CNS of human and mouse mainly secret the Th1-linked cytokines (such as IL-2 and IFN-γ), but not IL-4 (Kostic et al., 2015; Raphael et al., 2015). Furthermore, administration of IFN-γ to the patients with MS patients exacerbated the disease, whereas administration of neutralizing antibodies against IFN-γ had therapeutically beneficial effects (Kostic et al., 2015; Raphael et al., 2015).

The results of the current study also showed that the non-stimulated and MOG-stimulated PBMCs of the patients with MS expressed lower levels of *GATA-3* than those of the equal cell cultures from healthy individuals. These findings indicate that the down-regulation of Th2 cell-associated responses may be involved in the MS pathogenesis. *GATA-3* performs a key role in the Th2 cell differentiation, while suppressing Th1 cells (Zhang et al., 2014b).

In agreement with the current study results, it was demonstrated that transgenic mice over-producing *GATA-3* reduces Th1 cell-mediated inflammatory reactions in the CNS, as well as very low clinical signs, after EAE induction (Fernando et al., 2014). Moreover, epidemiological investigations in humans indicate that the patients with allergic asthma, a Th2 cell-related disorder, had much less risk for MS (Pedotti et al., 2009). In addition, the beneficial effects of glatiramer acetate (currently used to treat MS) may be partially performed through increasing Th2 cell-related immune response, which could reduce the deleterious Th1 cell-related responses (Oreja-Guevara, Ramos-Cejudo, Aroeira, Chamorro, & Diez Tejedor, 2012). In EAE models, the disease recovery was also related to the upregulation of Th2 cell-related cytokines in the brain (Kostic et al., 2015). Furthermore, the induction of Th2 cell-associated immune response resulted in the delay in the onset and severity of EAE (Fernando et al., 2014; La Flamme et al., 2006).

Th2 cells can provide protective effects against MS through the induction of the alternative (M2) type of macrophages/microglia (Kostic et al., 2015; Raphael et al., 2015). M2 type of macrophages/microglia is characterized by the lack of MHC II molecules, high capability of myelin phagocytosis, production of anti-inflammatory cytokines (such as TGF-β, IL-10, and IL-1 receptor antagonist), high expression of arginase-1 (an enzyme with capacity to inhibit activated microglia), low expression of the enzymes involved in generation of Reactive Oxygen Species (ROS) (Kostic et al., 2015; Raphael et al., 2015).

The current study revealed that the *T-bet*/*GATA-3* expression ratio in non-stimulated, MOG-stimulated, and PHA-stimulated PBMCs of patients with MS were higher than those of the same cell cultures from the healthy individuals. Therefore, an imbalance in Th1/Th2-related responses with a deviation toward Th1 cell may be involved in the pathologic process of MS. Since the *T-bet*/*GATA-3* expression ratio is considered as a useful alternative parameter to determine Th1/Th2 cells status, the current study results support a Th1-biased pattern in patients with MS, which was consistent with data from other investigations in patients with MS (Kostic et al., 2015; Raphael et al., 2015).

The maintenance of the normal immunological functions depends on a balance between Th1 and Th2 cell-related responses. On the other hand, the imbalance of Th1/Th2 cells may cause a number of disorders such as cancer, autoimmunity, allergic and infectious diseases (Zhang et al., 2014a). The current study also found that the *T-bet* expression increased, whereas the *GATA-3* expression decreased and hence the *T-bet*/*GATA-3* expression ratio rose by non-stimulated and stimulated PBMCs of the patients with MS. Therefore, an imbalance in the Th1/Th2 cells with a tendency toward Th1 cell occurs at the transcription factor level in patients with MS.

The current study results also indicated no significant differences between females and males regarding the *T-bet* expression in not-stimulated and stimulated PBMCs, neither in the healthy individuals nor in the patients with MS. Accordingly, the *T-bet* expression was not influenced by gender. It was also found that the *T-bet* expression increased, while the *GATA-3* expression diminished and therefore the *T-bet*/*GATA-3* expression ratio increased in non-stimulated and stimulated PBMCs of male and female patients with MS as compared with those of the same cell cultures from healthy individuals with the same gender. Accordingly, the upregulation of Th1 cell and/or downregulation of Th2 cell-related immune responses (the Th1/Th2 imbalance) may be involved in the pathogenesis of MS in both genders.

The results of the current study also demonstrated that the non-stimulated, MOG-stimulated, and PHA-stimulated PBMCs of male patients with MS expressed higher amounts of *GATA-3* when compared with those of the same cell cultures of females with MS. However, the *T-bet*/*GATA-3* expression ratio in MOG-stimulated and PHA-stimulated PBMCs of females with MS were higher than those of the counterpart cell cultures in male patients with MS. Therefore, the gender of patients with MS may influence the *GATA-3* expression and *T-bet*/*GATA-3* expression ratios. In accordance with the current study findings, it was found that the PBMCs of female patients with MS produced higher amounts of IFN-γ in response to myelin-originated proteolipid protein when compared with those of the control female and male patients with MS. In addition, when the survival rate was adjusted in relation to general mortality rates, female patients with MS expressed lower survival rate (Ngo, Steyn, & McCombe, 2014).

In conclusion, these results indicate that the *T-bet* expression increases, while the *GATA-3* expression decreases and thus the *T-bet*/*GATA-3* expression ratio increases in non-stimulated, MOG-stimulated, and PHA-stimulated PBMCs of the patients with MS. These results may indicate an imbalance in Th1/Th2 cells at the level of transcription factor and a Th1 cell biased pattern in patients with MS. Moreover, the *GATA-3* gene may be differently expressed in male and female patients with MS. The clinical utilization of the transcription factors as novel biomarkers of MS should be evaluated in further studies.

## Ethical Considerations

### Compliance with ethical guidelines

The local Ethics Committee of Kerman University of Medical Sciences approved the study protocol. The written informed consent was also obtained from each participant.
